# Early Life Stress and Metabolic Plasticity of Brain Cells: Impact on Neurogenesis and Angiogenesis

**DOI:** 10.3390/biomedicines9091092

**Published:** 2021-08-26

**Authors:** Alla B. Salmina, Yana V. Gorina, Yulia K. Komleva, Yulia A. Panina, Natalia A. Malinovskaya, Olga L. Lopatina

**Affiliations:** 1 Research Institute of Molecular Medicine & Pathobiochemistry, Professor V.F. Voino-Yasenetsky Krasnoyarsk State Medical University, 660022 Krasnoyarsk, Russia; yana_20@bk.ru (Y.V.G.); malinovskaya-na@mail.ru (N.A.M.); 2 Laboratory of Brain Cytology, Division of Brain Research, Research Center of Neurology, 125367 Moscow, Russia; 3 Department of Biochemistry, Medical, Pharmaceutical & Toxicological Chemistry, Professor V.F. Voino-Yasenetsky Krasnoyarsk State Medical University, 660022 Krasnoyarsk, Russia; yuliakomleva@mail.ru (Y.K.K.); yulia.panina@list.ru (Y.A.P.); ol.lopatina@gmail.com (O.L.L.)

**Keywords:** brain plasticity, neurogenesis, cerebral angiogenesis, mitochondria, glycolysis, brain metabolism, early life stress

## Abstract

Early life stress (ELS) causes long-lasting changes in brain plasticity induced by the exposure to stress factors acting prenatally or in the early postnatal ontogenesis due to hyperactivation of hypothalamic-pituitary-adrenal axis and sympathetic nervous system, development of neuroinflammation, aberrant neurogenesis and angiogenesis, and significant alterations in brain metabolism that lead to neurological deficits and higher susceptibility to development of brain disorders later in the life. As a key component of complex pathogenesis, ELS-mediated changes in brain metabolism associate with development of mitochondrial dysfunction, loss of appropriate mitochondria quality control and mitochondrial dynamics, deregulation of metabolic reprogramming. These mechanisms are particularly critical for maintaining the pool and development of brain cells within neurogenic and angiogenic niches. In this review, we focus on brain mitochondria and energy metabolism related to tightly coupled neurogenic and angiogenic events in healthy and ELS-affected brain, and new opportunities to develop efficient therapeutic strategies aimed to restore brain metabolism and reduce ELS-induced impairments of brain plasticity.

## 1. Introduction

Early life stress (ELS) is a well-known phenomenon underlying long-lasting changes in brain plasticity caused by the exposure to stress factors acting prenatally or in the early postnatal ontogenesis [1]. Various prenatal or early postnatal stimuli can result in the initiation of mechanisms specific for ELS, i.e., hypoxia, toxic agents, aberrant social interactions. It is well-established [2,3] that multiple physiological mechanisms are affected in ELS, thereby leading to impairment of brain development, neurological deficits, and higher susceptibility to development of aging-associated neurodegeneration in latter periods of life. This is known as an early programming phenomenon, or priming of developing brain to pathological conditions due to experiencing the prenatal, intranatal, or early postnatal stress [4]. Stress response is always associated with hyperactivation of hypothalamic-pituitary-adrenal axis (HPA) and sympathetic nervous system, establishment of pro-inflammatory mechanisms, and long-lasting epigenomic changes that affect normal development of brain cells, multicellular ensembles, and neural circuits [4,5,6,7,8]. In addition, ELS results in dramatic metabolic changes leading to insulin resistance, obesity, and metabolic syndrome [9,10]. However, some data suggest that ELS my increase resistance to other types of chronic stress in adolescence [11], but such data should be carefully interpreted as a result of diverse vulnerability of various animal strains to the action of ELS or application of different stressor protocols [12].

Neurogenesis, synaptic turnover and remodeling of neural circuits are the basis of brain plasticity in developing, mature, and ageing brain [2,13]. ELS affects brain developmental program, embryonic and adult neurogenesis, as well as cerebral angiogenesis that are tightly coordinated in normal conditions. These processes demonstrate significant alterations caused by ELS either due to inappropriate development of neurovascular niches (NVNs)—subventricular zone (SVZ) and hippocampal subgranular zone (SGZ), or remarkable changes in the local niche microenvironment induced by high levels of stress hormones and cytokines at the critical and sensitive periods of brain development [14,15]. The common finding is that ELS induces suppression of adult neurogenesis and augments the susceptibility to development of cognitive deficits or depression later in life [2]. However, there are important issues that should be considered in this context. For instance, female mice exposed to ELS at postnatal days 2–9 (P2–9) exhibit mild cognitive changes and no reductions in hippocampal neurogenesis, but male mice show impaired cognition and significant reduction in neurogenesis [16]. Paradoxically, ELS at P2–9 increases developmental neurogenesis in mice hippocampus, but reduces dentate gyrus volume and survival of adult-born neurons by P150 in males but not in females, thereby suggesting that long-lasting changes in brain plasticity caused by aberrant neurogenesis persist predominantly in the male brain [17]. Not only classical (SGZ, SVZ) neurogenic niches are affected by ELS: the number of stem and proliferating cells in the adult hypothalamus (subependymal niche) is also reduced by ELS exposure [18]. Recent findings demonstrate that if ELS exposure coincides with the period of stress hyporesponsivity with low corticosterone levels (first two weeks of postnatal life in rodents), neurogenesis might be enhanced either in the hippocampus or in the amygdala, but excessive neurogenesis leads to worsening the memory parameters, probably due to phenomenon of neurogenesis-driven hippocampal clearance [19].

ELS-induced deregulation of neurogenesis and cerebral angiogenesis in the immature brain might result in the development of numerous mental disorders. Schizophrenia links to the ELS pathogenesis via altered neurogenesis, presence of microvascular abnormalities, and deregulated brain development, presumably due to hypoxia-driven mechanisms [20]. Perinatal/neonatal hypoxia may result in excessive cell proliferation in particular brain regions and the elevated expression of schizophrenia risk genes [21,22,23]. Activity of several angiogenesis-related signaling pathways is down-regulated in the brain of patients with schizophrenia that demonstrate vascularization abnormalities [24,25]. Alzheimer’s disease is another example of ELS-driven alterations in neurogenesis/angiogenesis leading to postponed changes in brain plasticity: chronic distress increases the risk of dementia development [26] mainly due to neurogenesis impairments in wild-type mice, but not in transgenic Alzheimer’s disease model mice [27]. This is associated with defective pericyte coverage of microvessels and blood-brain barrier (BBB) breakdown in the prefrontal cortex, development of microglia-driven neuroinflammation, and cognitive dysfunction [28]. Developmental dyslexia results from the ELS-induced deregulation in the stress response system, epigenetic changes, and alterations in the establishment of brain asymmetry [29]. At the same time, this pathology is associated with abnormal neurogenesis and cell migration leading to aberrant cognitive development in humans [30], and deficiency of the vascular endothelial growth factor (VEGF) [31]. Thus, deregulated neurogenesis and angiogenesis/barriergenesis are pivotal mechanisms of ELS-mediated brain pathology.

While talking on ELS-driven impairment of brain plasticity, the following issues should be taken into consideration: (i) stress should be defined as any environmental stimulus which exceeds the physiological regulatory capacity of an organism and cannot be controlled efficiently [15]; (ii) effects of stress on neurogenesis and angiogenesis might be contradictory—enhancement, suppression, or deregulation—probably because of variable sensitivity at different stages of ontogenesis, or due to gender-specific response: maternal deprivation results in opposite changes in hippocampal neurogenesis in male and female offspring [14,32]; (iii) angiogenesis and neurogenesis are intrinsically coupled mechanisms in the developing and mature brain: vascular scaffold supports neural stem cells (NSCs) and neural progenitor cells (NPCs) maintenance, proliferation, differentiation, and migration of their progeny, whereas NSCs/NPCs stimulate establishment of new cerebral microvessels [33,34]; (iv) consequences of ELS-altered neurogenesis might be quite different in rodents and in mammals with big-sized brains because of unequal contribution of adult-born immature neurons within the neurovascular niches (NVNs) or non-newly generated immature cortical neurons (nng-Ins) to the brain plasticity [35]; (v) ELS-mediated aberrant embryonic angiogenesis results in BBB breakdown, development of neuroinflammation associated with cognitive and behavioral deficits later in life [36]; (vi) neurobehavioral alterations—anxiety, depression, aberrant social recognition, cognitive dysfunction—are common consequences of ELS [37,38]; (vii) metabolism of brain cells contributing to neurogenesis and angiogenesis might serve as a target for restoration of brain plasticity affected by ELS [36,39,40]. The latter conclusion is based on a huge volume of data on significant changes in the brain metabolic status in ELS and ELS-driven pathologies [10,41,42].

In this review, we will mainly focus on brain mitochondria and energy metabolism related to neurogenic and angiogenic events in healthy and ELS-affected brain, and new opportunities to develop efficient therapeutic strategies aimed to restore brain metabolism and reduce ELS-induced impairments of brain plasticity.

## 2. Developmental Aspects of Brain Metabolism and Effects of ELS

It is commonly accepted that the brain utilizes up to 20–25% of the body’s energy due to high demand in ATP for maintaining the critical activities of brain cells (information processing, neuronal excitation, cell-to-cell communications, neurites growth, local immune response, etc.). Normally, the main source of energy in the brain is glucose used in mitochondrial respiration, which dominates in neurons, or in glycolysis in glial cells [43,44]. In the resting adult brain, the oxygen–glucose index (OGI) is close to 5.5, whereas brain activation results in transient uncoupling of glycolysis and OXPHOS (OGI is 5.0) due to active glycolysis [43,45]. In early childhood, OGI is 4.1 again due to enhanced activity of glycolysis, and it reflects extensive brain plasticity, synaptic remodeling, neurogenesis, and brain growth in this period [46,47]. In the aging brain, there is a loss of glycolytic activity, probably due to the reduction of synaptic plasticity and neotenous processes seen in ageing [48]. Under starved conditions, the brain is re-profiled to use ketone bodies (synthesized in the liver and astrocytes). The same is true for developing prenatal or early postnatal brain, which prefers lactate and ketone bodies for energy production, and where both these metabolites are used as signaling molecules [49,50,51,52].

Within the neurovascular unit (NVU), the following mechanisms reflect the efficacy of energy metabolism in different cell types: (i) neuron-astroglial and astrocyte-endothelial metabolic coupling: active neurons stimulate astrocytes to produce lactate, cerebral endothelial cells elevate the lactate transport to supply this metabolite to neurons for its rapid conversion into pyruvate and ensuring the functioning of neuronal tricarboxylic acid (TCA) cycle or mitochondrial electron transport chain; (ii) gliovascular control: increase in the local concentrations of extracellular lactate leads to vasodilation, which is required for adjusting the blood flow and oxygen/nutrients supply to fit the metabolic needs of active brain regions; (iii) metabolic control of BBB structural integrity by brain microvessel endothelial cells (BMECs) that are highly enriched in mitochondria, and endothelial progenitor cells (EPCs) recruited from the bone marrow to establish new microvessels; (iv) establishment of pro-neurogenic and pro-angiogenic microenvironment with the niche hypoxia stimulating glycolysis and corresponding changes in NSCs/NPCs metabolism; (v) metabolic control of axons myelination: high mitochondria activity in oligodendrocyte progenitor cells is later replaced by glycolytic shift in mature oligodenroglia; (vi) metabolic control of neuroinflammation: reactive microglia requires extensive glycolysis for phagocytosis, migration, and production of cytokines. Key metabolic characteristics of NVU cells are summarized in Table 1.

In the first 2–3 weeks of postnatal development in mice (it corresponds to approx. 5 years of postnatal human development), brain glucose metabolism and oxygen consumption as well as the cerebral blood flow are 3–7 times higher than in subsequent periods of life. This period is characterized by active turnover of synapses, high electrical activity in cortical neurons. Exactly during this time-frame, there is a 4-fold increase in the number of mitochondria, mitochondrial proteins, and activity of mitochondrial respiratory chain in brain cells [79]. Later throughout life, the brain prefers to consume glucose, which is transported into the brain tissue via insulin-sensitive GLUT4 or partially insulin-sensitive GLUT1 glucose transporters expressed in BMECs within the NVU/BBB [80]. Actually, the main difference in the metabolic plasticity of cells in the developing and mature brain is that the ability to diversify sources for energy production is significantly higher at the earliest stages of ontogenesis, probably, due to the progressive age-dependent decrease in the expression of the corresponding transporters (i.e., monocarboxylate transporters, MCTs) in the BBB [52].

**Figure 1:**
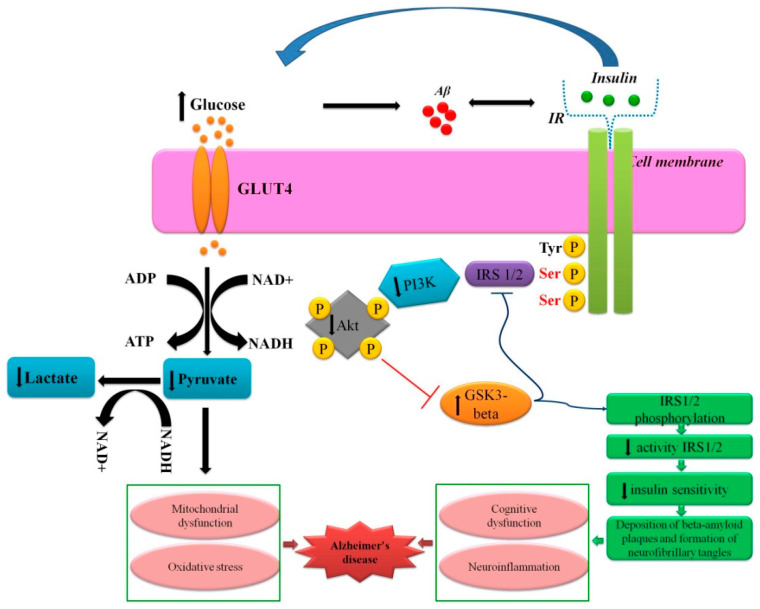
Insulin signaling in the brain in Alzheimer’s disease. Accumulation of Aβ disrupts the binding of insulin to insulin receptors on brain cells leading to aberrant IRS1/PI3K signaling, especially at the level of IRS1. It decreases PI3K/Akt activity and increases GSK-3beta kinase activity, which promotes phosphorylation of tau protein and increased deposition of Aβ-consisting plaques as two key hallmarks of Alzheimer’s disease. Reduced uptake of glucose through GluT transporters and intracellular hypometabolism of glucose in the brain cells (diminished glycolysis and OXPHOS) caused by insulin resistance are associated with the development of mitochondrial dysfunction, ROS overproduction, and oxidative damage of biomacromolecules.

The pre-existing opinion that the brain is an insulin-independent organ has been questioned by the hypothesis that insulin-mediated signal transduction is important for brain plasticity by regulating intercellular communication, energy homeostasis, growth and survival of neurons, and synaptic plasticity. Particularly, insulin-stimulated glucose metabolism is confirmed with PET protocols: brain glucose uptake and metabolism seems to be insulin-sensitive or operating at the top of the dose-response curve at basal peripheral insulin concentrations [80]. Activation of insulin receptors in brain cells in the olfactory bulbs, cortex, hippocampus, hypothalamus, and amygdala results in phosphorylation of insulin receptor substrate (IRS) protein, activation of phosphatidylinositol 3-kinase (PI3K), protein kinase B (Akt), and inhibition of glycogen synthase 3β kinase (GSK3β). These events lead to significant changes in neuronal metabolism like elevated glucose utilization and conversion to lactate, facilitation of mitochondrial oxidative phosphorylation (OXPHOS) [81]. Thus, it is not surprising that aberrant insulin signaling and brain glucose hypometabolism are considered as components of the pathogenesis of Alzheimer’s disease and progression of physiological aging: these metabolic phenomena trigger a cascade of pathological events, namely mitochondrial dysfunction, oxidative stress, excitotoxicity, apoptosis, and activation of pro-inflammatory cytokines [81,82,83,84] (Figure 1). Recently, systemic insulin resistance and development of diabetes mellitus in ageing mice (P350) that underwent ELS in the form of maternal separation at the neonatal period was clearly demonstrated [85], but whether these data might be extrapolated on the cerebral mechanisms of insulin signaling remains unclear.

Among all the NVU cells, neurons and BMECs are especially enriched in insulin receptors and mitochondria, whereas glial cells metabolism attributes to maintaining high basal levels of lactate. In neurons, perisynaptic mitochondria play an important role in the control of neuronal excitability, but contribution of astrocytes located in tripartite synapses is also high. Upon excitation, OXPHOS in mitochondria of neuronal dendrites is always supported by the subsequent active glycolysis in astrocytes to fuel the TCA cycle in neurons via MCT-mediated transport of lactate from astrocytes [86]. Even astrocyte–neuron metabolic coupling based on direct lactate transfer from astrocytes into neuronal cells has been debated in recent years; the role of lactate as a gliotransmitter and a signaling molecule in the brain is no longer disputed [87]. Moreover, astrocyte–neuron metabolic coupling has been shown to be involved in the complex mechanisms of learning, long-term memory consolidation, and even stress coping behavior [88,89]. Astrocyte–neuron lactate shuttle mechanism is complemented with the transfer of toxic oxidized fatty acids from activated neurons to astroglia [90] to prevent neuronal death caused by overexcitation, and with the transport of lactate via MCT transporters expressed in endothelial cells [91]. Astroglia-derived lactate affects other NVU/BBB cells, i.e., BMECs may respond to lactate either by activating lactate influx through MCT1 or by initiating signal transduction from lactate GPR81 receptors expressed on their plasma membrane. Experimental data suggest that both these mechanisms are required for maintaining the integrity of BBB as well as for effective angiogenesis/barriergenesis, and they are altered in neuroinflammation and ELS [2,69,92,93,94].

Metabolism of cells with neurogenic (NSCs/NPCs) and angiogenic potential (EPCs, tip- and stalk BMECs) is different from mature post-mitotic brain cells or other vascular cells. Particularly, quiescent endothelial cells (ECs) use glycolysis and fatty acids oxidation (FAO) to support their activity [95], but stimulation of angiogenesis results in insulin-dependent increase in glucose oxidation in tip-cells, activation of OXPHOS, fatty acids oxidation (FAO) and glycolysis in stalk-cells [96]. Within the neurogenic niches, NSCs/NPCs survival and self-renewal are driven by extensive glycolysis and mitochondrial FAO, whereas OXPHOS, excessive mitochondrial ROS production, corresponding changes in mitochondrial morphology, and elevated lipogenesis are activated when these cells are recruited to neurogenesis [97,98]. It is interesting to note that abnormal activity of some of these metabolic processes results in evident neurodevelopmental alterations. It was shown that inborn defects of FAO is a prerequisite for aberrant neurogenesis seen in autism [99], presumably, because FAO supports stem cells survival in metabolic stress [100]. At the same time, extremely high levels of FAO inhibit glycolysis by means of accumulation of acetyl-CoA, NADH, and inhibition of pyruvate dehydrogenase (PDH), thereby leading to mitochondrial dysfunction and elevated ROS production [101].

ELS affects metabolism in all the tissues: deregulation of glucose utilization and energy production leads to increased risk of metabolic syndrome, diabetes mellitus, cardiorenal syndrome, and obesity [102]. In mice fed a standard diet, maternal separation which is a well-known established model of ELS increases fasted blood glycemia, induces glucose intolerance and decreases insulin sensitivity in postnatal day 350 (P350) [85]. ELS-induced insulin resistance and metabolic syndrome were detected in monkeys [9] and humans [103].

It is commonly accepted that the main stress-induced change in the cellular metabolism is the altered balance of glycolytic to mitochondrial production of ATP. In prenatal stress in rats, glycolysis is increased whereas OXPHOS is decreased in the brain of 3-month-old offspring [104]. However, dysfunctional mitochondria in brain cells increase stress susceptibility along with compromised neurogenesis in the mouse model of stress-induced depression [105]. The duration of ELS could dictate the pattern of metabolic changes that would result in secondary alterations. Particularly, acute stress induces increase in the cerebral levels of extracellular lactate [106] followed by the reduction of glucose metabolism [107]. Such changes stem from the stress hormones action, subsequent metabolic adaptation, development of insulin resistance, and epigenetic reprogramming [108,109,110]. No doubts, mitochondria play the central role in the stress response by means of intracellular control of multiple signaling pathways, apoptosis, autophagy, reactive oxygen species, and energy production [43,111]. Mitochondria might modulate the individual effects of stress and HPA activity as it was demonstrated in mice that harbored different mitochondrial gene variants and subjected to the psychological stress [112]. Thus, the balance of glycolytic and mitochondrial energy production would control the cell stress response as well as HPA responsivity in ELS-affected organisms.

ELS-induced metabolic alterations result in paradoxical acceleration of cell maturation confirmed with the markers of synaptic maturity and myelination, and earlier development of emotional behavioral patterns [113]. In general, the brain development program is altered by ELS: long-lasting alterations in social behavior and hippocampal expression of neurotransmitter receptors have been registered in adult mice underwent ELS up to P17 [114], mitochondrial dysfunction and increased number of immature spines in hippocampal neurons have been found in juvenile 28-day-old mice with ELS [115], anxiety-like behavior and aberrant hippocampus-dependent memory have been detected later in the life of rats exposed to ELS at P2–P14 [116].

Presumably, it might be caused by global glucose hypometabolism and reduced activity of glycolysis in the developing brain affected by ELS, therefore, biosynthetic activity and synaptic plasticity are reduced. Indeed, decreased levels of glutamate, γ-aminobutyrate, N-acetylaspartate, glutamine, and phosphorylcholine that might be considered as markers of neurodegeneration or neurodevelopmental disorders like autism or schizophrenia have been detected in the brain of rodents exposed to maternal separation [41].

Taking into consideration the above-mentioned data, one may assume that insulin resistance is a key mechanism of long-lasting changes in the brain metabolism induced by ELS [42]. Risk of Alzheimer’s type neurodegeneration is much higher in ELS-affected individuals [117]. Progression of Alzheimer’s type of dementia often associates with local insulin resistance [118]; thus, it should be considered as a factor contributing to higher vulnerability of brain to development of Alzheimer’s disease later in the life. All these changes are preceded by acute stress-induced alterations in brain metabolism that mainly focus around the increase in glucose utilization and lactate overproduction aimed to support neuronal activity and neurotransmitter synthesis in stress conditions [41]. Since reduced levels of neurotransmitters were detected 2–5 months later after the maternal separation, lactate-driven mechanism of adaptation to the acute stress was already non-functional to support biosynthetic activity of mature neurons. However, in PET assessment of cerebral glucose metabolism in adult monkeys that have experienced ELS being infants, enhanced metabolism of glucose was shown in superior temporal sulcus, putamen, thalamus, and inferotemporal cortex in ELS animals compared to the controls [119].

Mitochondrial functional activity and dynamics are significantly affected by ELS: maternal separation in pups later results in lower ability of animals to perform Morris water maze, and such abnormalities correspond to deregulated activity of cytochrome *c* oxidase. Its elevated activity was found in the cingulate cortex, anterior thalamus, and supramammillary area, but diminished activity was demonstrated in medial mammillary nucleus [120]. Comparative proteomic and functional analysis of hippocampus in juvenile and adult male and female mice that underwent ELS reveals that there is an enrichment in proteins associated with mitochondria and OXPHOS in females, increased OXPHOS in juvenile males and females, but reduced OXPHOS in adult females [121]. Thus, gender- and age-dependent mechanisms are activated by ELS exposure, thereby resulting in a wide spectrum of outcomes later in life. It is known that ELS frequently results in development of depression (maternal separation is used as a validated model of depression in rodents), so, mice with deficiency of Complex I in mitochondria have been proposed as a suitable model of stress-induced phenomenon of early life programming leading to various behavioral manifestations, including depression [105]. Moreover, mitochondrial dynamics in neurons of nucleus accumbens reflects expression of depression-like behavior in mice, and mitofusins regulating mitochondrial fusion have been proposed as candidate target molecules for the treatment of depression and anxiety [122].

The simplest explanation of mitochondrial alterations in ELS is stress hormones-induced dysfunction of mitochondria, resulting in the suppression of respiration, development of Ca^2+^ imbalance, oxidative stress and apoptosis [123]. However, it might have no relation to the postponed changes in mitochondria structure developed after ELS. So, what might be a mechanism of ELS-induced long-lasting changes in mitochondria activity? Presumably, it relates to epigenetic changes in mitochondrial or nuclear DNA leading to altered mitochondrial dynamics or diminished activity of mitochondrial enzymes. Such proposal has got some arguments in recent experimental studies and was excellently reviewed in [124]. Particularly, early-life social isolation results in elevated levels of neuronal H3K9me2, which corresponds to reduced expression of NMDA receptor subunits, NR1, and AMPA receptor subunits, GluR1 and GluR2 in rat hippocampus [125]. Methylation of BDNF gene was found to be altered in the prefrontal cortex of mice exposed to ELS [126]. However, the pattern of epigenetic modifications caused by ELS depends on the specific type of ELS model applied (i.e., social isolation vs. social stress) since different brain regions might be affected [127]. Dual-activation hypothesis was proposed to connect the activity of HPA and sensory networks at particular critical developmental periods with transient or permanent epigenetic changes induced by ELS: epigenetic reprogramming of HPA is completed due to changes in the expression of genes encoding stress hormones or neuropeptides (i.e., glucocorticoid hormone and its receptors, arginine vasopressin and its receptors, oxytocin and its receptors) in brain cells. For instance, changes in the DNA methylation of a gene promoter site and histone acetylation would result in altered binding of transcription factors and modified expression of a corresponding gene, thereby leading to prominent changes in the responsivity of HPA axis in the conditions of acute stress [128]. At the level of neural networks, epigenetic reprogramming is a result of aberrant gene expression in NSCs/NPCs and their progeny caused by DNA methylation and demethylation known to affect activity-dependent neurogenesis [129]. Synaptic experience-driven plasticity is under the control of DNA methylation and histone modifications [130]. Thus, prenatal and juvenile postnatal periods known as sensitive phases of brain development are compromised by significant and long-lasting changes in HPA responsivity and functional integration of newly-formed neurons [131]. Later in life, this could lead to development of stress-related brain disorders, accelerated aging, and neurodegeneration.

How might mitochondria be involved in such epigenetic mechanisms? From one side, mitochondria produce huge number of metabolites that affect nuclear and mitochondrial epigenetic machinery: FAD, NAD^+^, acetyl–CoA and α-ketoglutarate of mitochondria origin are associated with the processes of active methylation/demethylation or acetylation/deacetylation of DNA histones [132]. In the case of mitochondrial DNA, modification of histones is not possible because of their absence there, but mitochondrial DNA itself is a target for methylation/demethylation occurring predominantly at non-CpG sites, which result in long-lasting or even permanent changes in mitochondrial activity [133,134,135]. In addition, the presence of mitochondrial DNA in a close vicinity to ROS produced in mitochondria matrix leads to accumulation of nucleoside 8-hydroxy-2-deoxy-guanosine (8-OHdG) and impairment of mtDNA transcription/replication [136]. Thus, epigenetic mechanisms of long-lasting changes in mitochondrial metabolism in ELS-effected brain are plausible. However, it is still rather difficult to clearly distinguish the molecular mechanisms of metabolic events that are caused by acute stress and those developed in the affected brain later in life.

## 3. Neurogenesis and Cerebral Angiogenesis: Search for a Common Regulator of Metabolic Reprogramming

It is commonly accepted that cognitive reserve depends not only on the efficacy of neurogenesis or synaptogenesis [137], but also on adequate neurovascular coupling: human fMRI studies demonstrate that hippocampal vascularization pattern modulates cognitive performance and hippocampal integrity, thereby suggesting that mixed blood supply from both the posterior cerebral artery and the anterior choroidal artery is most effective to support cognitive functions [138]. Thus, it is reasonable that under the conditions of enhanced demand for neurogenesis (e.g., learning), suppression of cerebral angiogenesis results in the impairment of learning and memory [139]. Exercise-induced angiogenesis in brain tissue is well-documented phenomenon and might be partially responsible for cognition-enhancing effect of regular physical activity [140]. Also, it is known that post-ischemic angiogenesis contributes to brain tissue repair and neuronal remodeling via metabolic support of developing neuronal cells, guidance of cells and axons toward the ischemic locus, and oxygen supply to the neurogenic niches for initiating proliferation and differentiation of cells [141].

*Vascular control of neurogenesis: supply of oxygen and regulatory molecules*. In the whole brain, hippocampal vascularization seems to be reduced compared with neocortex due to increased capillary spacing in hippocampus, specific (less-contractile) morphology of hippocampal pericytes, and low ability of hippocampal BMECs to promote vasodilation [142]. Thus, it is not surprising that hippocampal cells survive in less availability of oxygen comparing to cortex cells, and low oxygen concentrations exist in neurogenic niches that are important for maintaining the pool of NSCs/NPCs [143]. Hypoxia prevents recruitment of NSCs/NPCs and increases their survival, whereas local hyperoxia stimulates proliferation and differentiation of embryonic cells in vitro [143,144]. That is why hyperbaric oxygen therapy promotes neurogenesis in post-stroke rats in vivo [145]. When hypoxia within neurogenic niche is reduced by newly-formed microvessels, NSCs diminish their glycolysis and start to differentiate in the embryonic brain [146]. In this context, it is reasonable that functional hyperemia in the adult dentate gyrus promotes experience-induced hippocampal neurogenesis required for efficient learning, as was demonstrated in [147].

**Figure 2:**
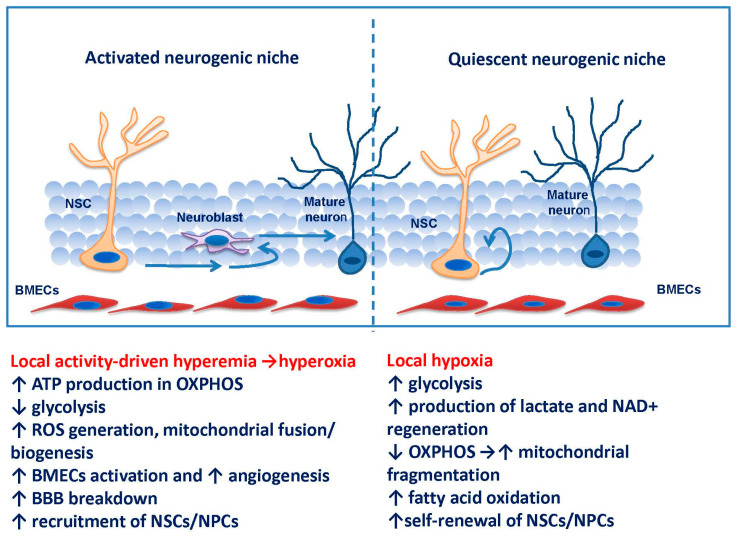
Metabolic reprogramming in NSCs/NPCs associated with conversion from the quiescent into activated state. Neurogenic niche is represented here as a simplified model. In a quiescent niche, relatively low oxygen tension keeps the cells out of cell cycle. Activation of neurogenesis by physiological stimuli (e.g., learning) is associated with functional hyperemia and local BBB breakdown to provide better access to pro-neurogenic factors, and with the switch from glycolytic to mitochondrial ATP production associated with mitochondrial biogenesis and oxidative stress to support proliferation and differentiation of cells.

Since neoangiogenesis is functionally coupled to neurogenesis, it is tempting to speculate that in “dormant” neurogenic niches in hippocampus, basal level of blood supply results in relatively low local oxygen tension, suppression of mitochondrial ATP generation and corresponding changes in mitochondrial dynamics (mitochondrial fragmentation), elevated production of lactate in glycolysis, and self-renewal of NSCs/NPCs. This is quite different from the severe hypoxia caused by global brain ischemia, which stimulates recruitment of NSCs/NPCs, probably due to elevated levels of extracellular lactate [148], thereby contributing to reparative neurogenesis. In the case of experience-driven brain plasticity (e.g., learning), local functional hyperemia leads to the stimulation of mitochondrial fusion and biogenesis, increased ATP production, and proliferation and differentiation of NSCs/NPCs and their progeny to support learning and memory [143,145]. These events are associated with reversible and partial BBB breakdown to provide better access of regulatory molecules to the niche, and elevated endothelium-mediated transport of lactate [91] (Figure 2).

Memory formation is associated, upon encoding, with a hypoxic signal triggering transitory cerebral angiogenesis in specific cortical regions aimed to support memory storage and retrieval some weeks later [149]. Therefore, one may assume that hypoxia-driven cortical neoangiogenesis might be responsible for the so-called hippocampal-cortical memory trace transfer [150] and mechanism of hippocampal clearance to minimize the interference of old and new memories [151]. Thus, conversion of a hippocampal neurogenic niche from the quiescent to the activated state might be coupled with transient local vasodilation and BBB breakdown, whereas memory consolidation in the cortex requires extensive neurogenesis and neoangiogenesis.

The above-mentioned events reflect the situation of metabolic reprogramming, which is a widely-spread phenomenon related to the ability of cells to change their metabolism on demand [152]. Mammalian cells often face situations when they have to switch their metabolism to the most preferable mode of functioning. For instance, neural stem and progenitor cells advance the way of proliferation-differentiation-migration: proliferation and self-renewal require extensive glycolysis, lowered OXPHOS and mitochondria fragmentation, whereas differentiation means activation of OXPHOS and mitochondria elongation, finally resulting in development of mature post-mitotic neurons enriched in mitochondria [153,154,155]. Such dramatic metabolic changes vary in cells that are naïve or primed [156], but according to the commonly accepted view, NSCs/NPCs have higher glycolytic requirements than mature neurons [157]. Thus, the question arises: what are the key regulators of metabolic reprogramming of cells with pro-neurogenic or pro-angiogenic potential? One may propose that such regulatory molecules should have direct relation to the main metabolic pathways that contribute to ATP production; however, their involvement in other biochemical processes, i.e., those related to amino acid metabolism, could be considered as well [156].

*Glycolysis- and OXPHOS-(re)generated NAD^+^.* One of the factors controlling the mechanism of metabolic reprogramming is the activity of pyruvate dehydrogenase (PDH): its activation means generation of acetyl-CoA fueling TCA cycle, whereas its inhibition means activation of pyruvate–lactate conversion in glycolysis. Activity of PDH is negatively regulated by insulin-driven C-Jun N-terminal kinase (JNK), and this effect is abolished by protein kinase C (PKC) [158]. More generally, this means that a cell can increase the availability of NAD^+^ for numerous metabolic events (activity of NAD^+^-dependent enzymes, NAD^+^-consuming enzymes) by reducing PDH activity. Indeed, regeneration of NAD^+^ accompanies pyruvate-to-lactate conversion as well as activity of the mitochondrial electron–transport chain. In mammalian cells, metabolism of NAD^+^ is required for various enzymes controlling proliferation and differentiation: poly(ADP-ribosyl)polymerase (PARP) serving for DNA replication and repair, mono(ADP-ribosyl)transferases affecting cell signaling, histone deacetylases/sirtuins regulating gene transcription, and NAD^+^-glycohydrolases synthesizing second messengers with Ca^2+^ mobilizing activity (cyclic ADP-ribose or nicotinic acid adenine dinucleotide phosphate) [159,160,161,162].

Thus, if a cell actually needs more NAD^+^ than ATP, activation of glycolysis is preferred [163]. Since self-renewing/proliferating stem cells are under permanent replication stress, they always need high levels of intracellular NAD^+^ to support PARP activity for efficient DNA repair [164]. Therefore, stem cells keep glycolytic flux at the high level, and they are well-equipped with the machinery for efficient NAD^+^ synthesis and metabolism. For instance, ablation of nicotinamide phosphoribosyl transferase (NAMPT)—the key enzyme for NAD^+^ synthesis—in NSCs/NPCs reduces the pool and proliferation of these cells in the hippocampus of mice [165]. In human embryonic stem cells, NAD^+^ increases mitochondrial oxidative metabolism, partially suppresses glycolysis, stimulates amino acid turnover, doubles the consumption of glutamine, and these effects are coupled with the expression of markers of pluripotency and proliferation, thereby suggesting that NAD^+^ is required for self-renewal and prevention of differentiation of stem cells [166]. In human mesenchymal stem cells, decrease of NAD^+^/NADH ratio results in cellular senescence and loss of sirtuin1 activity [167]. Supplementation of NAD^+^ with its precursors prevents stem cells senescence [167], rejuvenates aged intestinal stem cells [168], and delays senescence of NSCs in mice [169]. Thus, it is rather reasonable that restoration of NAD^+^ levels supports prenatal cerebral angiogenesis and improves postnatal behavioral phenotype [36], stimulates adult neurogenesis [170], prevents neurodegeneration [171], and supports stress response [172]. Preservation of NAD^+^ levels with precursors of NAD^+^ (e.g., nicotinamide mononucleotide) prevents age-dependent decline in cerebral angiogenesis and reduces endothelial oxidative stress in rats [173]. NAD^+^ deficiency in pregnant rodents results in congenital malformations in offspring and miscarriage [174], thus suggesting the importance of keeping the adequate levels of NAD^+^ for normal development. Promotion of adult neurogenesis in offspring could be achieved by NAD^+^ supplementation (using nicotinamide riboside as NAD^+^ precursor) of mice mothers in the postpartum period, probably, through transition of NAD^+^ with mother milk to pups [175]. Moreover, behavioral abnormalities (social deficits and anxiety) seen in CD157 knockout mice can be efficiently corrected with nicotinamide riboside replenishing the intracellular NAD^+^ levels needed for oxytocin release and establishment of appropriate social (re)cognition [176].

Switching from glycolysis to mitochondrial respiration in brain cells is associated with extensive mitochondrial biogenesis. It is interesting that mitochondrial dynamics itself affects neurogenesis: in embryonic cortical neurogenesis in mice, mitochondrial fusion redirects cells towards self-renewal status in NAD^+^- and sirtuin1-dependent manner [177]. The same might be true for angiogenesis: in endothelial tip-cells migration, activation and differentiation are supported by glycolytic flux and mitochondrial fragmentation [67,178], but proliferation of endothelial stalk-cells depends on balanced combination of glycolysis and OXPHOS [179]. Activation of microglia or astroglia in the loci of neuroinflammation is associated with the metabolic switch from OXPHOS to glycolysis (similar to Warburg effect) to provide support for cytotoxicity and cytokines release [180]. All these changes are accompanied by reciprocal mitochondrial dynamics: fission/fragmentation of mitochondria or even mitophagy occurs in cells with predominant glycolysis, whereas fusion of mitochondria or mitochondrial biogenesis are important for cells with elevated demand for OXPHOS and other homoeostatic events in mitochondria (production of ROS, Ca^2+^ release and Ca^2+^ uptake, synthesis of steroids, synthesis of heme, etc.) [181]. Since mitochondrial biogenesis is driven by sirtuin1-PGC-1-dependent mechanisms [182], one may assume that high intracellular levels of NAD^+^ required for sirtuin1 activity control the establishment of new functional mitochondria and balance of self-renewal and differentiation capacity of NSCs/NPCs as well as EPCs/endothelial stalk-cells. However, some studies revealed that increase in intracellular NAD^+^/NADH ratio and activation of sirtuin1 could lead to mitochondrial fragmentation and mitophagy in fibroblasts [183], thereby suggesting variability in mitochondrial dynamics-mediated processes in different cell types or in various conditions.

Recent data reveal that chronic prenatal stress in mice suppressed expression of NAD^+^ synthesis enzymes associated with abnormal brain cortical GABA interneuron development, axonal degeneration in the hippocampus, progression of cognitive deficits and depression-like behavior in offspring [184]. It is well-known that any stress leads to depletion of intracellular NAD^+^ levels due to development of mitochondrial dysfunction, overproduction of ROS, oxidative damage of DNA, and hyperactivation of NAD^+^-consuming enzyme PARP, which further inhibits glycolysis, thereby leading to energetic catastrophe and cell death [185]. Theoretically, cells may combat such conditions by activating AMP kinase (AMPK) able to stimulate NAD^+^ biosynthesis via salvage pathways [186]. However, since expression of NAD^+^ synthesis enzymes is disrupted by prenatal stress [184], this mechanism would be non-functional in the ELS-affected brain. At the same time, AMPK can stimulate mitochondrial biogenesis in a PGC-1-dependent manner [187]; thus, this effect might be very relevant. Indeed, PGC-1 levels are decreased in the brain of adult offspring rats that underwent prenatal stress [188], but stimulation of PGC-1-driven mitochondrial biogenesis is efficient in reducing the negative consequences of prenatal hypoxic-ischemic brain injury in rats [189]. Thus, bioavailability of NAD^+^ might be a key regulatory factor in brain cells metabolic reprogramming. If so, stress-driven depletion of intracellular NAD^+^ could lead to suppression of PDH, metabolic switch between glycolysis and OXPHOS, and aberrant activity of NAD^+^-converting enzymes. Numerous examples of mitochondrial programming caused by ELS in rodents and humans have been analyzed in detail [190,191], but it is still unclear whether such changes contribute to altered neurogenesis and cerebral angiogenesis in early life.

*Neurotransmitters derived from the TCA cycle metabolites.* Glutamate which may undergo transamination to α-ketoglutarate to support the TCA cycle is one of the most important excitatory neurotransmitters, whereas GABA is synthesized from glutamate due to activity of glutamic acid decarboxylase (GAD) to provide inhibitory neurotransmission in the adult brain [192]. In immature neurons, GABA acts as an excitatory neurotransmitter, but in the postnatal mature brain, it is the main inhibitory neurotransmitter. Such excitatory (depolarizing) effect of GABA on neural progenitors is required for their maturation [193]. In the adult hippocampal dentate gyrus, local parvalbumin interneurons are also depolarized by GABA, which is necessary for keeping NSCs in quiescent conditions and preventing the depletion of NSCs pool [194,195]. It should be noted that newly synthesized and integrated hippocampal neurons respond to the action of GABA as immature ones (depolarization) and they gradually become hyperpolarized along their functional and morphological maturation, whereas the depolarizing effect of glutamate is critical for their competitive survival and synaptic integration [196]. GABA regulates neurogenesis [197], partly, due to BMECs-derived GABA, which supports neuronal migration [198]. Thus, two neurotransmitters derived from the TCA cycle contribute to the regulation of cell fate in the developing brain or in neurogenic niches of adult brain. Whether or not PDH activity could dictate the balance of depolarizing and hyperpolarizing signals in stem/progenitor or neuronal cells requires further assessment.

The exact mechanism of such regulation relates to the phenomenon of excitation/inhibition (E/I) balance: stem and progenitor cells serve as targets for the action of GABA in neurogenic and oligovascular niches [199]. As is shown, the ratio of excitatory and inhibitory conductance (E/I balance) is required for preventing inappropriate response of neurons to input strength; this mechanism matures at the early postnatal period (P8-P18 in mice) due to changes in the expression of chloride transporters NKCC1 and KCC2 induced by intranatal action of oxytocin [199,200]. Thus, it was proposed that one of the main goals of adult neurogenesis might be the restoration of E/I balance in the pre-existing neural circuits of hippocampus [199,201].

ELS induced by maternal separation in rats accelerates synaptic activity in hippocampus; thus, LTP peaks in adolescence (P22–24) but not in adulthood [202], and age-dependent shift in spontaneous excitatory synaptic currents relative to inhibitory synaptic currents was significantly accelerated by ELS in mice [203]. ELS-induced changes in E/I balance are not specific for hippocampus, since neurons in the prefrontal area [204] and infralimbic prefrontal layer II/III [203] are affected as well. In sum, E/I balance is greatly affected in ELS, thereby contributing to the changes in stem and progenitor cells proliferation and differentiation.

*Bioactive lipids.* Recent experimental findings suggest that changes in E/I balance in the activated hippocampus (hippocampus-dependent contextual exploration) might relate to the metabolism and activity of sphingosine-1-phosphate (S1P) whose local signaling through sphingosine-1-phosphate receptor 2 (S1PR2) was increased to provide suppression of excitation and survival of newborn dentate gyrus cells [205]. Currently, S1P is considered as one of the candidates for the key regulators of adult neurogenesis and angiogenesis, and one may assume that it contributes to some of the ELS-mediated changes in brain plasticity. S1P is a sphingolipid which may elicit its effects either extracellularly or intracellularly being synthesized in the pathway from sphingomyelin to ceramide and sphingosine due to activity of membrane-bound or cytosolic sphingosine kinases, respectively [206]. Extracellular activity of S1P is mediated by receptors S1PR1–5 that are activated in paracrine or autocrine manner in various cells. Being expressed in the brain tissue, S1PR1 has been reported to take part in controlling the differentiation of brain endothelial cells, embryonic and adult angiogenesis and vascular maturation, maintenance of endothelial barrier structural and functional integrity by strengthening tight junction machinery; S1PR2 regulates excitation of pyramidal neurons and increases endothelial barrier permeability, thereby allowing development of neuroinflammation; S1PR5 controls proliferation of oligodendrocytes [207,208,209,210]. Recent data suggest that S1P might be important for the regulation of mitochondrial dynamics. In ECs, S1PR2 is involved in the high glucose-induced fission and fragmentation of mitochondria [211,212], thus, it is tempting to speculate that such activity might underlie S1PR2-driven BBB breakdown. Mitochondria may produce S1P due to sphingosine kinase 2 whose activity seems to be indispensable for mitochondrial respiration [213]. Intracellular targets for S1P are mitochondria, endoplasmic reticulum (ER), and various enzymes, i.e., β-site amyloid precursor protein (APP) cleaving enzyme-1, thereby, it directly regulates autophagy and proteolysis of intracellular proteins in neuronal cells [214,215]. Thus, taking into consideration numerous effects of S1P in brain cells, it is not surprising that deregulation of S1P metabolism and signaling attributes to pathogenesis of neurodevelopmental, neuroinflammatory, and neurodegenerative disorders [216]. However, involvement of S1P metabolism or activity in the brain development or in the pathogenesis of ELS remains unclear and requires further studies.

Experimental data confirm that S1P may act as an universal regulator of neurogenesis and angiogenesis within neurogenic niches. S1P action results in proliferation of neural progenitor cells in vitro that express S1PR1,2,3,5 [217]. Embryonic neural progenitor epithelial cells are sensitive to stimulatory action of S1P [218], and changes in the expression of S1PR1 leads to shift in the direction of migration of neuroblasts in vivo [219]. Application of FTY720, which acts on S1PRs, leads to enhanced migration of NPCs transplanted into mice, thus showing its potential as neurogenesis-promoting drug [220]. S1PR1 and S1PR3 mediate stimulatory action of S1P on ECs, resulting in their proliferation and migration [221]. However, some data taken from S1pr1−/− mice suggest that activation of S1PR1 results in inhibition of angiogenic sprouting [222].

As we discussed before, oxygen tension within the neurogenic niche controls the fate of NSCs/NPCs. In erythrocytes, S1P may act as a hypoxia-responsive lipid which promotes glycolysis [223], but it might be not true for mitochondria-containing cells. Indeed, S1P stimulates mitochondrial respiration in cardiac cells [213], promotes mitochondrial biogenesis in Hep G2 cells acting through PGC-1 [224], but negatively affects OXPHOS and mobilization of endogenous lipids to fuel FAO in T cells [225]. Thus, it might be hypothesized that production of S1P in NSCs/NPCs or accessory cells within neurogenic niches might coordinate metabolic reprogramming associated with the recruitment of stem cells. Particularly, basal levels of sphingimyelin-ceramide-S1P metabolism would result in suppression of OXPHOS to keep the pool of stem and progenitor cells, but activation of S1P production could lead to proliferation and migration of cells. At the same time, S1P stimulates establishment of new microvessels and BBB whose integrity would depend on the predominant expression of S1PRs on BMECs. However, whether or not this mechanism is functional in neurogenic niches in the brain remains to be evaluated.

## 4. Conclusions and Further Prospects: Strategies Aimed to Restore Metabolic Plasticity of Brain Cells for Efficient Neurogenesis and Angiogenesis

**Figure 3:**
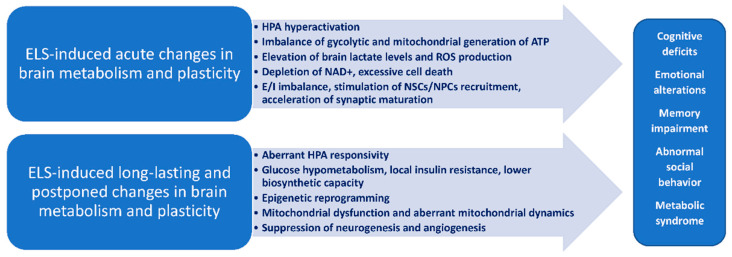
Acute and long-lasting/postponed changes in brain metabolism and plasticity induced by ELS.

There is growing evidence that metabolic status of brain cells controls brain plasticity and vulnerability to the action of stimuli affecting brain development [226], and it was clearly demonstrated in various physiological conditions or brain diseases, including those caused by ELS (Figure 3). Diversity of metabolic characteristics of brain cells at various stages of ontogenesis and in different brain regions, in immature or mature states, in active or resting conditions, suggests that it might be quite difficult to figure out the unified “metabolic pattern” of the brain activity. Thus, metabolism, in general, or mitochondrial dynamics and energy metabolism cannot be attributed to the whole brain tissue or even some brain regions, because it is an integral parameter derived from many types of cells with different activities [227].

However, numerous pharmacological agents that might affect energy production, mitochondrial dynamics and mitochondrial quality control in the tissue have been already tested in experimental conditions or even proposed for their application in clinical practice: dynasore and cilnidipine as inhibitors of mitochondrial fission, leflunomide as promoter of mitochondrial fusion, p53 and TSPO (mitochondrial translocator protein) inhibitors, iron chelator deferiprone, resveratrol as modulators of mitophagy, glycolysis inhibitors, nicotinamide, nicotinamide riboside and NAMPT activators as NAD^+^ boosting molecules [228,229,230,231]. Pharmacological agents that have been suggested for preventing or reducing the negative consequences of ELS, i.e., inhibitors of fatty acid amide hydrolase and cyclooxygenase-2, antidepressants with evident pro-neurogenic activity, methionine, valproic acid, theophyllin, fluoxetine and trichostatin A as epigenome-modulating agents, VEGF as neurogenesis- and angiogenesis-promoting factor, agents affecting oxytocinergic mechanisms, as well as enriched environment, are able to attenuate some neuropathological changes induced by ELS [232,233,234,235]. However, most of them have not been systematically tested as drugs or factors affecting brain cells metabolism, particularly, in the context of metabolic reprogramming of proliferating and differentiating cells within the neurogenic/neurovascular niches.

Presumably, focusing on the exact population of brain cells with similar functional competencies will allow deciphering the key biochemical and physiological signatures corresponding to particular regimens of brain activity in ELS. In this context, assessment of metabolic status and mitochondrial activity/dynamics in cells with confirmed neurogenic and angiogenic potential (NSCs, NPCs, EPCs, tip- and stalk-endothelial cells) either in vitro or in vivo is of great importance for the identification of molecular targets sensitive to pharmacological agents and drug candidates [236,237].

Analysis of current data on the role of metabolic reprogramming of stem and progenitor cells involved in neurogenesis and cerebral angiogenesis during their development reveals the following approaches that might have prospects in translational studies and clinical applications: (i) modulation of mitochondrial activity and glycolytic flux in target cells; (ii) modulation of mitochondrial dynamics, intracellular mitochondrial redistribution and intercellular mitochondrial transfer; (iii) manipulations with the intracellular NAD^+^ levels; (iv) modulation of cell-to-cell and intracellular signaling mediated by common metabolites; (v) modulation of neurovascular effects and local permeability of BBB for adequate supply of nutrients, oxygen, metabolites, and regulatory molecules, utilization of hypoxia/hyperoxia-driven metabolic control of neurogenesis and angiogenesis; (vi) modulation of E/I balance in the loci of neurogenesis and angiogenesis; (vii) differential targeting of neurogenic cells residing in conventional neurogenic niches (SGZ, SVZ), non-classic neurogenic niches (hypothalamus, amygdala, cerebellum, etc.), or non newly-generated DCX+PSA-NCAM+ immature neurons in the cortex, as well as of angiogenic cells (bone-marrow-derived EPCs, BMECs), and, probably some circulating multipotent cells of bone marrow origin for manipulating with developmental and experience-dependent brain plasticity. This is a new and exciting chapter in exploring the mechanisms of brain development and searching for effective ways to prevent the negative effects of early life stress on cognition and behavior.

## Figures and Tables

**Table 1:** Metabolic profile of brain cells.

Type of Cells	Metabolic Characteristics	References
Neurons	Oxidative phosphorylation (OXPHOS) dominates. Perisynaptic localization of mitochondria. Under stress and aging, they redistribute mitochondria in the cell and demonstrate impairment in glycose utilization and decline in mitochondrial function. Poorly utilize fatty acids.	[53,54,55,56]
Astrocytes	Glycolysis dominates. They support the metabolism of neurons with lactate. Capable of depositing the glycogen. Mitochondrial activity determines the efficiency of glutamate uptake. Serve as donors of mitochondria when neurons are damaged.	[57,58,59,60]
Oligodendrocytes	Myelination is under the control of OXPHOS (to a greater extent) and glycolysis, at the end of the myelination program, glycolysis dominates, and lactate maintains axonal vitality.	[61,62,63]
Microglia	Resting microglia uses OXPHOS, activated microglia uses glycolysis. Microglia activation is accompanied by mitochondrial fragmentation.	[64,65,66]
BMECs	Have a higher content of mitochondria than endothelial cells in other tissues. Angiogenesis is accompanied by increased glycolysis and OXPHOS, fatty acid oxidation. Disturbances in mitochondrial dynamics are characteristic of damage to the BBB. Various reactive oxygen species (ROS)-generating enzymes are expressed.	[67,68,69,70,71]
Pericytes	Glycolysis dominates; however, these cells might donate mitochondria for damaged perivascular astroglia.	[72]
NSCs/NPCs	Self-maintenance and proliferation of NSCs/NPCs require glycolysis, oxidation of fatty acids. Differentiation is accompanied by the prevalence of OXPHOS, and significant changes in the shape of mitochondria from fragmented to elongated. Able to deliver functional mitochondria to target cells. Aging NSCs/NPCs demonstrate lower oxidative metabolism.	[73,74,75,76,77,78]
